# First report of *Theileria* and *Anaplasma* in the Mongolian gazelle, *Procapra gutturosa*

**DOI:** 10.1186/s13071-014-0614-3

**Published:** 2014-12-21

**Authors:** Youquan Li, Ze Chen, Zhijie Liu, Junlong Liu, Jifei Yang, Qian Li, Yaqiong Li, Qiaoyun Ren, Qingli Niu, Guiquan Guan, Jianxun Luo, Hong Yin

**Affiliations:** State Key Laboratory of Veterinary Etiological Biology, Key Laboratory of Veterinary Parasitology of Gansu Province, Lanzhou Veterinary Research Institute, Chinese Academy of Agricultural Sciences, Xujiaping 1, Lanzhou, Gansu 730046 PR China; Jiangsu Co-innovation Center for Prevention and Control of Important Animal Infectious Diseases and Zoonoses, Yangzhou, 225009 People’s Republic of China

**Keywords:** *Theileria*, *Anaplasma*, Detection, *Procapra gutturosa*, PCR, China

## Abstract

**Background:**

*Theileria and Anaplasma* are especially important emerging tick-borne pathogens of animals and humans. Molecular surveys and identification of the infectious agents in Mongolian gazelle, *Procapra gutturosa* are not only crucial for the species’ preservation, but also provide valuable information on parasite and bacterial epidemiology.

**Findings:**

A molecular surveillance study was undertaken to assess the prevalence of *Theileria* spp. and *Anaplasma* spp. in *P. gutturosa* by PCR in China. *Theileria luwenshuni*, *A. bovis*, *A. phagocytophilum*, and *A. ovis* were frequently found in *P. gutturosa* in China, at a prevalence of 97.8%, 78.3%, 65.2%, and 52.2%, respectively. The prevalence of each pathogens in the tick *Haemaphysalis longicornis* was 80.0%, 66.7%, 76.7%, and 0%, respectively, and in the tick *Dermacentor niveus* was 88.2%, 35.3%, 88.2%, and 58.5%, respectively. No other *Theileria* or *Anaplasma* species was found in these samples. *Rickettsia raoultii* was detected for the first time in *P. gutturosa* in China.

**Conclusions:**

Our results extend our understanding of the epidemiology of theileriosis and anaplasmosis in *P. gutturosa*, and will facilitate the implementation of measures to control these tick-borne diseases in China.

## Findings

### Background

*Theileria* is mainly transmitted by tick vectors and cause heavy economic losses to the livestock industry. The family Anaplasmataceae in the order Rickettsiales was reclassified in 2001, and includes several genera, including *Anaplasma*, *Ehrlichia*, *Neorickettsia*, and *Wolbachia*. Of them, the genera *Anaplasma* and *Ehrlichia* are especially important as emerging tick-borne pathogens in both humans and animals [1]. *Anaplasma phagocytophilum* is the causative agent of human granulocytic anaplasmosis, an extremely dangerous disease associated with high mortality rates in humans [2-4]. Other *Anaplasma* spp., such as *A. bovis*, *A. ovis*, *A. marginale*, and *A. centrale*, infect the erythrocytes and other cells of ruminants [3,4]. Anaplasmosis is endemic in tropical and subtropical areas, but is also frequently reported in temperate regions. Six or seven *Anaplasma* species have been reported in North America, Europe, Africa, and Asia [5-11], and some have also been reported in sheep, goats, and cattle throughout China [9,12,13].

The detection and isolation of *Theileria* and *Anaplasma* require specialized laboratories staffed by technicians with a high degree of expertise, primarily because the species’ life cycles are intracellular. Several sensitive molecular tools, such as PCR, have been used to detect and identify *Theileria* and *Anaplasma* species in both hosts and vectors [10-17].

The Mongolian gazelle, an endemic ungulate species designated a threatened species by the World Conservation Union, is facing human and livestock disturbances of varying intensity in northern China. Although several studies have demonstrated that various *Theileria*, *Babesia*, *Ehrlichia*, and *Anaplasma* species circulate among sheep, goats, cattle, cervids, and humans in China, almost no data are available on the possible role of *P. gutturosa* as a host organism. The aim of this study was to detect and identify *Theileria* and *Anaplasma* spp. in *P. gutturosa*, a potential natural host of animal theileriosis and anaplasmosis in China.

## Methods

### Sample collection

The region investigated in China is located at latitudes 35°03′–35°55′ north and longitudes 105°37′–108°08′ east. The study was performed in April 2014. A total of 92 blood samples were collected randomly from *P. gutturosa*, and 242 ticks were collected from both *P. gutturosa* and grass in its environment. Of them, 30 unfed adult ticks were collected directly from grass in the gazelles’ environment; 212 engorged nymph ticks collected from *P. gutturosa* were kept at 28°C and 80-90% relative humidity during molt, until nymph ticks were molted into adult ticks. All of adults were identified with Teng’s methods [18]. Blood smears were prepared from the ear blood of every *P. gutturosa* individual. During the blood collection process, cases of suspected theileriosis or anaplasmosis were investigated. Theileriosis and/or anaplasmosis should be suspected in tick-infested animals with fever, enlarged lymph nodes (theileriosis only), anemia, and jaundice.

### Microscopic analysis of blood smears

**Figure 1:**
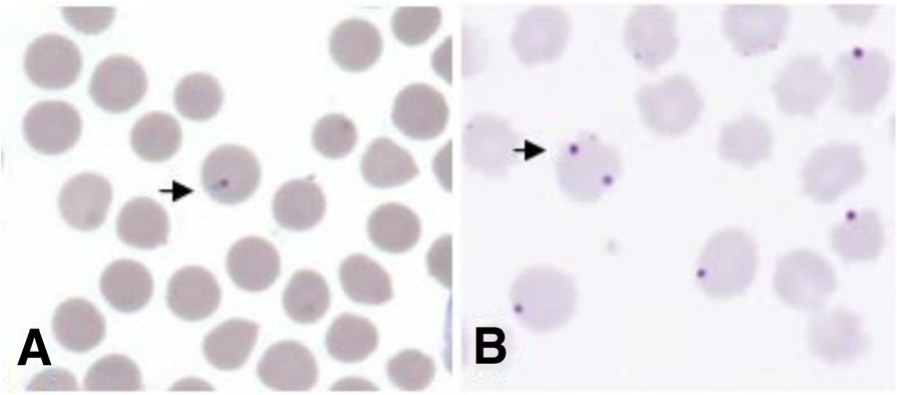
***Theileria*** **(A) and** ***Anaplasma*** **(B) in the blood smears from Mongolian gazelle.**

The blood smears were air-dried, fixed in methanol, stained with a 10% solution of Giemsa in phosphate-buffered saline (pH 7.2), and then analyzed microscopically and photographed (Figure 1).

### DNA extraction

Genomic DNA was extracted from the 92 whole blood samples and 222 tick samples using a genomic DNA extraction kit (Qiagen, Hilden, Germany), according to the manufacturer’s instructions. The DNA yields were determined with a NanoDrop 2000 spectrophotometer (Nanodrop Technologies, Wilmington, DE, USA).

### Molecular detection of *Theileria* and *Anaplasma* using species-specific primers

PCR was used to detect and identify *Theileria* and *Anaplasma* spp. in *P. gutturosa* with the species-specific primers shown in Table 1 [10,11,14-17]. The PCR reactions were performed in an automatic DNA thermocycler (Bio-Rad, Hercules, CA, USA) and the PCR products were used to assess the presence of specific bands indicative of *Theileria* and *Anaplasma*.

**Table 1:** **Sequences of the oligonucleotide primers used in this study**

**Pathogen**	**Target gene**	**Primers**		**Final amplicon size (bp)**	**References**
		**Primer name**	**Oligonucleotide sequences (5’-3’)**		
*Anaplasma & Ehrlichia*	16S rRNA	EC9	TACCTTGTTACGACTT	1462	Kawahara et al., 2006 [10]
		EC12A	TGATCCTGGCTCAGAACGAACG		
*A. bovis*	16S rRNA	AB1f	CTCGTAGCTTGCTATGAGAAC	551	Kawahara et al., 2006 [10]
		AB1r	TCTCCCGGACTCCAGTCTG		
*A. phagocytophilum*	16S rRNA	SSAP2f	GCTGAATGTGGGGATAATTTAT	641	Kawahara et al., 2006 [10]
		SSAP2r	ATGGCTGCTTCCTTTCGGTTA		
*A. marginale*	msp4	Amargmsp4 F	CTGAAGGGGGAGTAATGGG	344	Torina et al., 2012 [11]
		Amargmsp4 R	GGTAATAGCTGCCAGAGATTCC		
*A. ovis*	msp4	Aovismsp4 F	TGAAGGGAGCGGGGTCATGGG	347	Torina et al., 2012 [11]
		Aovismsp4 R	GAGTAATTGCAGCCAGGGACTCT		
Hemoparasite	18S rRNA	Primer A	AACCTGGTTGATCCTGCCAGT	1750	Medlin et al., 1988 [14]
		Primer B	GATCCTTCTGCAGGTTCACCTAC		
*Theileria*	18S rRNA	989	AGTTTCTGACCTATCAG	1100	Allosop et al., 1993 [15]
		990	TTGCCTTAAACTTCCTTG		
*T. luwenshuni*	18S rRNA	Tl310	GGTAGGGTATTGGCCTACTGA	340	Yin et al., 2008 [16]
		Tl680	TCATCCGGATAATACAAGT		
*Babesia*	18S rRNA	Babesia F	TGTCTTGAATACTT(C/G)AGCATGGAA	950	Ramos et al., 2010 [17]
		Babesia R	CGACTTCTCCTTTAAGTGATAAC		

The DNA fragments were sequenced by the GenScript Corporation (Piscataway, NJ, USA). Representative sequences of the 18S rDNA/16S rDNA (or *msp4*) genes of the *Theileria* and *Anaplasma* spp. newly identified in this study were deposited in the GenBank database of the National Center for Biotechnology Information (NCBI) (http://www.ncbi.nlm.nih.gov/genbank/).

### Sequence alignments and phylogenetic analyses

**Figure 2:**
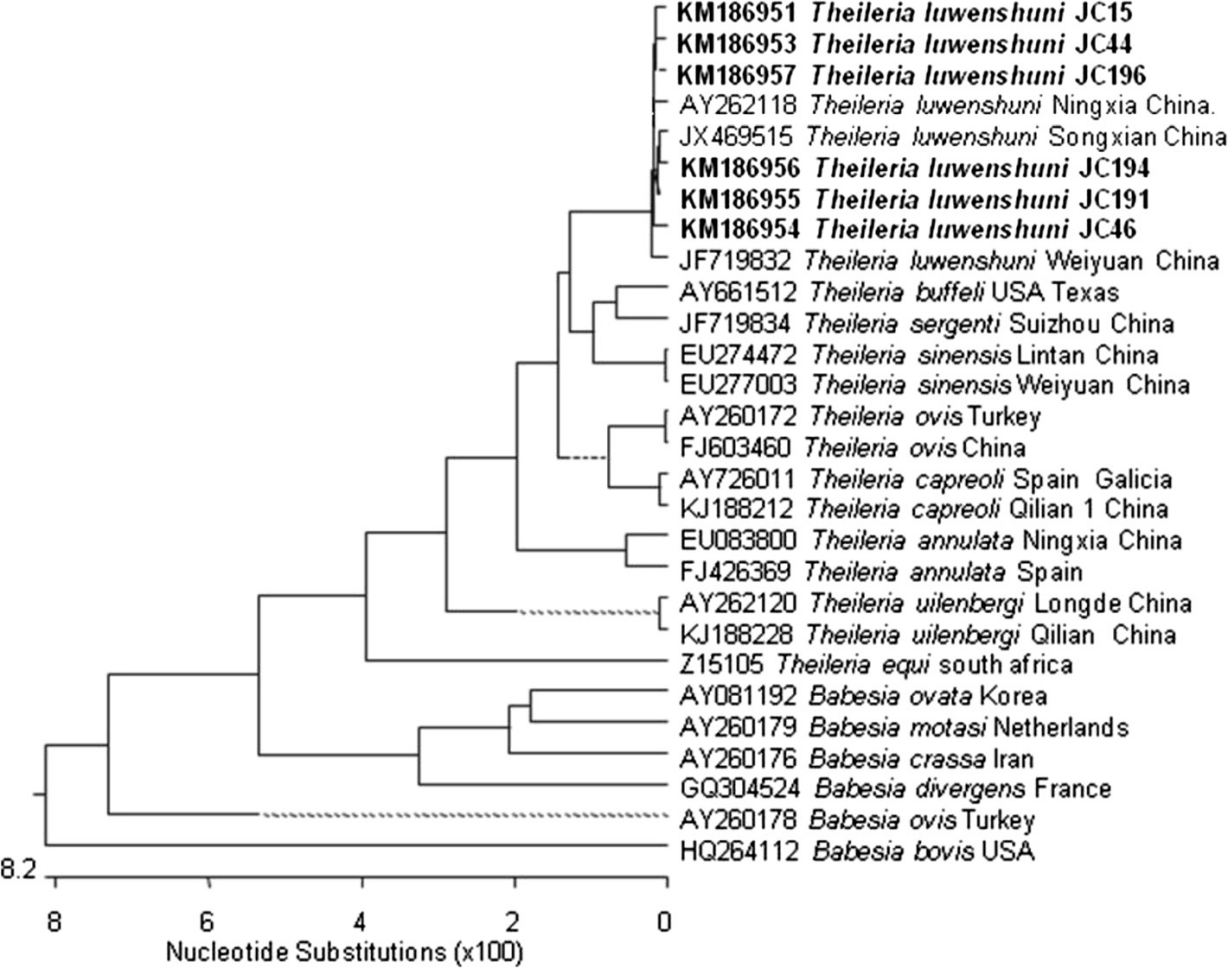
**Phylogenetic tree of** ***Theileria*** **and** ***Babesia*** **based on 18S rDNA sequences.**

**Figure 3:**
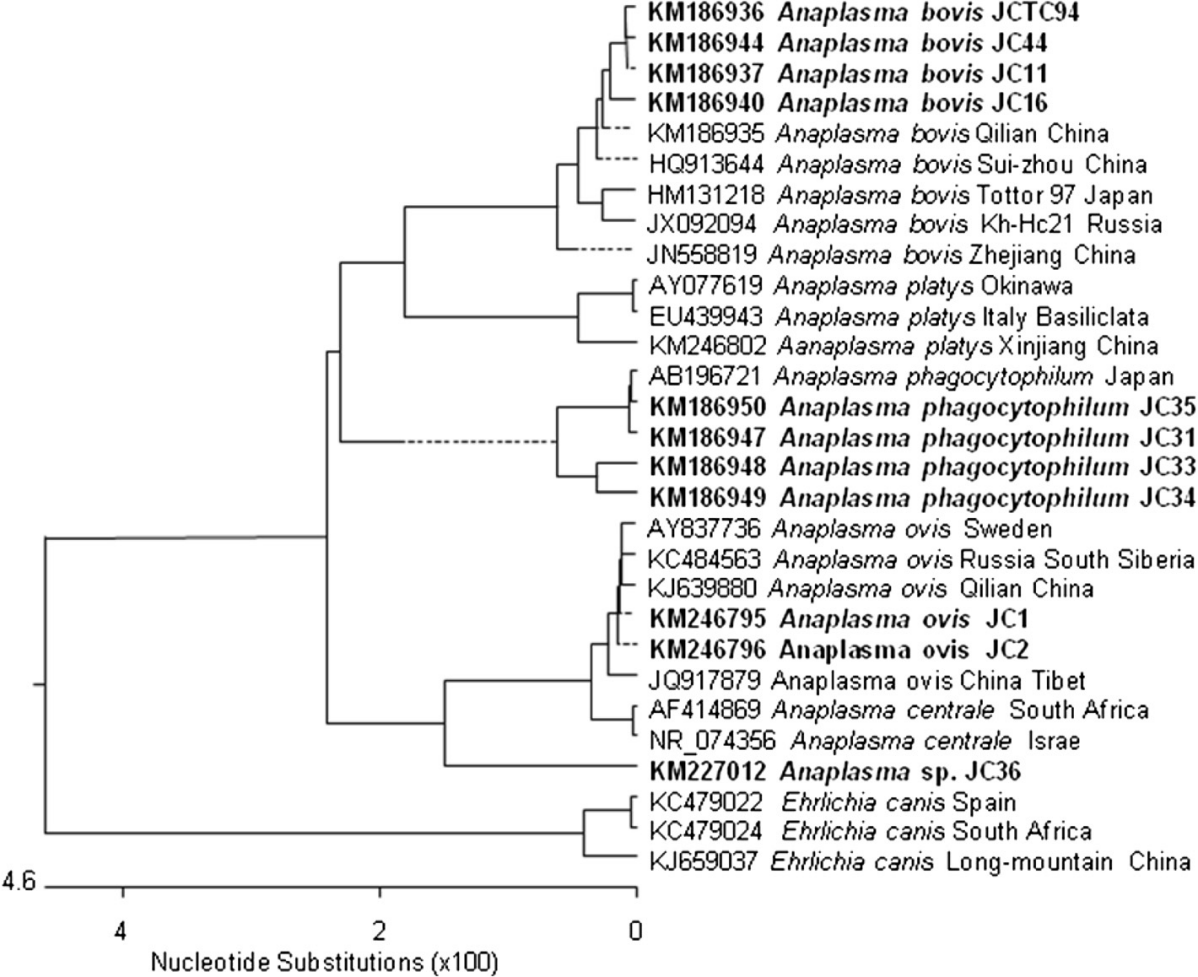
**Phylogenetic tree of** ***Theileria*** **and** ***Babesia*** **based on 16S rDNA sequences.**

The MegAlign component of the Lasergene® program version 4.01 (DNASTAR) was used to generate multiple sequence alignments with the ClustalW algorithm (www.clustal.org/) and for the phylogenetic analyses using the neighbor-joining method. A phylogenetic tree was constructed (Figure 2) based on the *Theileria* and *Babesia* 18S rDNA gene sequences determined in this study, and others obtained from the GenBank database under accession numbers: KM186951–KM186957, AY262118, JX469515, JF719832, AY661512, JF719834, EU274472, EU277003, AY260172, FJ603460, AY726011, KJ188212, EU083800, FJ426369, AY262120, KJ188228, Z15105, AY081192, AY260179, AY260176, GQ304524, AY260178, and HQ264112. Another phylogenetic tree was constructed (Figure 3) based on sequences of the *Anaplasma* and *Ehrlichia* 16S RNA genes under the following accession numbers: KM186935–KM186937, KM186940, KM186944, KM186947-KM186950, KM246795, KM246796, KM227012, HQ913644, HM131218, JX092094, JN558819, AY077619, EU439943, KM246802, AB196721, AY837736, KC484563, KJ639880, JQ917879, AF414869, NR_074356, KC479022, KC479024, and KJ659037.

### Ethical approval

This study was approved by the Animal Ethics Committee of Lanzhou Veterinary Research Institute, CAAS (No. LVRIAEC2013-010). The use of these field samples was approved by the Animal Ethics Procedures and Guideline of China.

## Results

### Tick identification

In this study, all 242 ticks were collected from *P. gutturosa* or grass in its environment in north-western China. The identification result showed that the adult ticks were either *Haemaphysalis longicornis* (n = 130: 86 female; 44 male) or *Dermacentor niveus* (n = 112: 78 female; 34 male). The whole DNA of 120 *H. longicornis* ticks and 102 *D. niveus* ticks was extracted.

### Microscopic examination of blood smears

Theileriosis and anaplasmosis was present in 50% of the gazelles tested (46/92). *Theileria* and *Anaplasma* infections were observed microscopically in 87.0% (80/92), and 13.0% (12/92) of the blood smears from *P. gutturosa* individuals, respectively (Figure 1). All infected animals exhibited low levels of parasitemia, with 0.01–6% for *Theileria* and 0.01–4% for *Anaplasma*.

### PCR detection of *Theileria* and *Anaplasma* with species-specific primer sets

PCR analysis revealed that the prevalence of *T. luwenshuni*, *A. bovis*, *A. phagocytophilum*, and *A. ovis* in *P. gutturosa* was 97.8%, 78.3%, 65.2%, and 52.2%, respectively. Their prevalence in *H. longicornis* was 80.0%, 66.7%, 76.7%, and 0%, respectively, and their prevalence in *D. niveus* was 88.2%, 35.3%, 88.2%, and 58.8%, respectively (Table 2). No *Babesia* sp. was found in *P. gutturosa*, *H. longicornis*, or *D. niveus*. Only one (4.3%) of the 92 samples from *P. gutturosa* was positive for *R. raoultii*.

**Table 2:** **Prevalence of** ***Theileria*** **and** ***Anaplasma*** **in** ***Procapra gutturosa*** **and ticks in China**

**Host**	**No. of samples**	**The prevalence of** ***Theileria*** **and** ***Anaplasma*** **in** ***Procapra gutturosa*** **and Ticks by PCR and Microscopic Examination**					
		**By Microscopic Examination (ME)**		**By PCR**			
		***Theileria*** **spp.**	***Anaplasma*** **spp.**	***T. luwenshuni***	***A. bovis***	***A. phagocytophilum***	***A. ovis***
*Procapra gutturosa*	92	87.0% (80/92)	13.0% (12/92)	97.8% (90/92)	78.3% (72/92)	65.2% (60/92)	52.2% (48/92)
*H. Longicornis*	120	/	/	80.0% (96/120)	66.7% (80/120)	76.7% (92/120)	0%
*Dermacentor niveus*	102	/	/	88.2% (90/102)	35.3% (36/102)	88.2% (90/102)	58.8% (60/102)

### Amplification of the 18S/16S rDNA or *msp4* genes and their accession numbers

The nearly full-length 18S rDNA sequences of *T. luwenshuni* were 1745 bp with the primers A/B, and the accession numbers are KM186951–KM186957. The nearly full-length 16S rDNA sequences were 1457 bp in *A. bovis* (KM186936–KM186944), 1458 bp in *A. phagocytophilum* (KM186947–KM186950), and 1456 bp in *A. ovis* (KM246795 and KM246796) with primers EC12/EC12A, which are specific for *Anaplasma* and *Ehrlichia* spp. An unknown *Anaplasma* sp. was isolated and its accession number was KM227012. The *msp4* gene PCR products were 551 bp for *A. bovis* (KM226988, KM226999, KM227002, and KM227003), 641 bp for *A. phagocytophylum* (KM227007–KM227009), and 347 bp for *A. ovis* (KM227005 and KM227006) when species-specific primers were used.

### Sequence alignments and phylogenetic analyses

The phylogenetic tree based on the *Theileria* and *Babesia* 18S rDNA sequences showed that only one pathogen was detected, which was placed in the *T. luwenshuni* cluster (Figure 2). The phylogenetic tree based on the 16S rDNA sequences of *Anaplasma* and *Ehrlichia* revealed four pathogens existed and they were *A. bovis*, *A. phagocytophilum, A. ovis*, and *Anaplasma* sp., respectively, in the blood samples from *P. gutturosa* roaming northern China (Figure 3).

## Discussion

To the best of our knowledge, this study is the first to report the prevalence of theileriosis and anaplasmosis in *P. gutturosa* in China. Molecular screening of *P. gutturosa* in northern China showed that the most prevalent *Theileria* and *Anaplasma* species were, in descending order: *T. luwenshuni* > *A. bovis* > *A. phagocytophilum* > *A. ovis*. No other *Theileria* sp. or *Anaplasma* sp. was detected in *P. gutturosa*. The prevalence of *T. luwenshuni* and *A. bovis* in *P. gutturosa* was higher than their prevalence in *H. longicornis* or *D. niveus*. However, the prevalence of *A. phagocytophilum* was, in descending order: *D. niveus* > *H. longicornis* > *P. gutturosa*. We speculate that persistent pathogen reservoirs with high infection rates are well established in *P. gutturosa* in northern China.

*Anaplasma bovis* infections of cattle have been reported predominantly in African countries, and there have been few reports of bovine *A. bovis* infections in China. Recently, *A. ovis* and *A. bovis* were reported in goats in central and southern China, and *A. marginale* was detected in cattle in southern China [9]. *A. bovis* and *A. ovis* have also been reported in red deer, sika deer, and *D. everestianus* in north-western China [12]. In Japan, *A. bovis* and *A. centrale* have been detected in wild deer and *H. longicornis* ticks on Honshu Island, Japan [10]; *A. bovis* and *A. phagocytophilum* were initially detected in cattle on Yonaguni Island, Okinawa, Japan [19]. Therefore, *H. megaspinosa* is considered a dominant vector tick species for both these species in cattle in Japan [20]. In this study, four *Anaplasma* spp. (*A. bovis*, *A. ovis*, *A. phagocytophilum*, and an *Anaplasma* sp.) were detected in *P. gutturosa. Rickettsia raoultii* was also detected for the first time in *P. gutturosa* in China.

In this study, all 242 ticks were collected from gazelle or from their environment in the investigated area. They consisted of *H. longicornis* and *D. vineus. Theileria luwenshuni* and *Anaplasma* spp. (including *A. bovis*, *A. phagocytophilum, A. ovis*, and *Anaplasma* sp.) were detected and identified by PCR. Therefore, we speculate that these ticks play an important role as natural vectors of *Anaplasma* spp. in northern China. *Theileria luwenshuni* were first reported in sheep and goats, and widely distributed in north-western China [21]; recently, it was also reported in sheep and goats in central and southern China [22-24]. *T. luwenshuni* can be transmitted by *H. qinghaiensis* and *H. longicornis* in north-western China [25], but only *H. longicornis* and *D. niveus* were found in this study. Therefore, *H. longicornis* must play an important role as a natural vector of *T. luwenshuni* in *P. gutturosa* in northern China*.* However, whether *T. luwenshuni* can be transmitted by *D. niveus* remains to be determined.

## Conclusion

Our results provide important data that extend our understanding of the epidemiology of theileriosis and anaplasmosis, and should facilitate the implementation of measures to control the transmission of *Theileria* and *Anaplasma* among *P. gutturosa* and other relative ruminants in China. Clarification of the role of *P. gutturosa* as a reservoir host for some *Theileria* and *Anaplasma* species is critical in determining whether *P. gutturosa* contributes to the spread of ruminant theileriosis and anaplasmosis in China.
